# Western life courses challenged in life stories collected in contemporary China

**DOI:** 10.3389/fpubh.2023.1282704

**Published:** 2023-12-05

**Authors:** Sheng-Li Cheng, Stina Johansson, Shushan Liu, Yun Li

**Affiliations:** ^1^School of Philosophy and Social Development, Shandong University, Jinan, China; ^2^Department of Social Work, Faculty of Social Sciences, Umeå University, Umeå, Sweden

**Keywords:** life stories, life courses, Chinese older adult, qualitative research, social transformation

## Abstract

**Introduction:**

The life course describes the progression of life as a social role, from birth to death. Traditional Western life-history studies of the life course usually describe a continuous lifeline with occasional interruptions in between. The extraordinary temporal events of the Anti-Japanese War (AJW), Civil War (CW), Great Famine (GF), Cultural Revolution (CR), and the Reform and Opening-up of China in just few decades mean that the life history of contemporary Chinese older adults may be quite different from those of their western peers.

**Methods:**

The study used qualitative research methods to conduct in-depth interviews with 16 older adults and collect their life stories through a standardized list of questions. Grounded theory was employed to condense, compare, conceptualize, and synthesize patterns within the collected data, approaching the investigation with a “naturalistic” perspective.

**Results:**

Based on a generalized analysis of the Interview transcripts, we can find that Chinese old adults’ life stories were shaped by recurring exceptional and rapidly changing environmental conditions. The themes and sub-themes of Chinese old adults’ life stories were focus on (1) violence, loss of family member, escape and unstable life in their early life which are related to AJW and CW; (2) poverty and starvation in daily life when they were adolescents and young adults which are related to GF; (3) discontinuity, timed opportunities, categorizations in order to split the social relationships and networks in CR. The data also suggest that education is an important part of the life story and that its value changes over time.

**Discussion:**

The discontinuity and instability of the life stories of the Chinese old adults are unexpected according to the dominant Western-influenced life course theories, which enriches life course theory and provides a new perspective for studying the individual life course in a society of constant and rapid change.

## 1 Introduction

The term ‘life course’ describes the progression of lives from birth to death as a sequence of social roles (1, 2). In conventional Western life-history research, a continual lifeline, with short discontinuity passages, is normally narrated. Early life living conditions have an influence on the different stages of life. A life course perspective provides an opportunity to see the diversity of how past conditions continue to affect the present situation of the older adult (3, 4) and how extraordinary conditions during a short period create a common base of experiences related to the cohort a person was born into. One study proposes a model for “building longevity over the life course” from the perspective of active aging and the life course (5). There is evidence for cumulative inequality over the life course [(6, 7); for an overview, see (8)]. Furthermore, interaction effects show that adverse living conditions strengthen the effect of early life on life satisfaction in later life.

**Figure 1:**
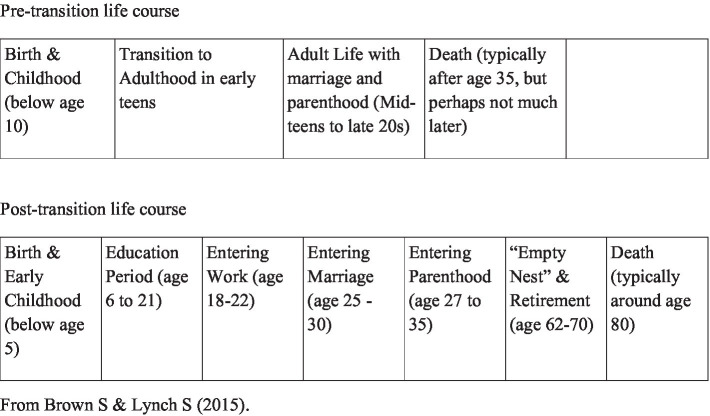
Hypothetical life course sequences and durations.

Western life-history research often divides life into different stages related to family and work (9) (see Figure 1). The passage between the different stages and how the next stage will look often exhibits a certain degree of predictability. People from Western countries are used to planning for a life with relatively stable periods punctuated by occasional interruptions, such as economic downturns. The stages of uncertainty and lack of structure are usually short. Recent studies have proposed a life course cube as a synthetic representation of the life course, in which the axes represent three dimensions of time, domains, and levels at which developmental, behavioral, and societal processes occur (10–13). The life course cube also defines the interdependencies of the three dimensions and their multiple interactions (14). The stability, as well as the predictability related to chronological age and fixed phases, has been questioned (15).

The historical time, China from the middle and later part of the 20th century, from which our informants have delivered some material for us to reflect over, includes narratives about a long series of more or less chaos. Old persons, who were born in the historical time when the Anti-Japanese War (AJW), Civil War (CW), Great Famine (GF), and Cultural Revolution (CR) came to affect their lives, tell us about their childhood and youth, the period that came to shape their circumstances in life and their identities. Some studies show that about 9 million Chinese civilians died in the Anti-Japanese War, and 95 million people became refugees [History (16)]; other studies show that totally about 20.6 million people died as a direct result of the war, plus disabled and missing people caused by the war, and casualties caused by Japanese army’s aggression to China were over 45 million. Millions of Chinese families were displaced, broken up, and bereaved (17). After the Anti-Japanese War, China entered a 4-year-long CW, until the establishment of the People’s Republic of China in 1949. During 1959–1961, China experienced serious economic difficulties (18). A widespread famine occurred, the living standards of people declined dramatically, a lot of people starved to death, and many families fled their homes to make a living.

From 1966 to 1976, China experienced a decade-long Cultural Revolution, and the state “send-down” policy in the People’s Republic of China forced 17 million urban youth to live and work in rural areas. It was a kind of “natural experiment” organized by the state. All social groups were negatively affected by adverse state policies. The send-down experience has had lasting effects on individuals’ life courses, as reflected in the patterns of the later life course events (19). Many families were ripped apart even with some family members dying. CR seriously undermined the political, economic, social, cultural, and ethical basis of the society. This period has been called “ten years of chaos” and “ten years of catastrophe” (18). Dikotter (20) describes it as a tumultuous era with unintended consequences. The decadal send-down mobilization in China has fundamentally changed the life course of a generation of urban youth. Their education was interrupted, and their marriage and childbearing were delayed. Zhou and Hou (19) show that those who had shorter send-down durations were significantly better off in later life course events, compared with those with longer durations. The legacy of the send-down episode is likely to continue to influence the life course of the children of the CR.

In the next 30 years, China has experienced both an economic transition from a planned economy to a market economy, and a social transition from a traditional agricultural society to an industrial society and even a post-industrial society. The proportion of older adult has increased faster than in any other country in the world, something that has caused huge changes in family structure and the expectations of intergenerational exchange (21).

Unique is that during a short period, so many extraordinary off-time events happened. A person born at the beginning of the years of birth that our material represents had quite other challenges in life than persons born some years later. Life expectations and possibilities to plan life were for many Chinese citizens limited.

A life course perspective emphasizes the variation of individual life courses as a consequence of historical and social arrangements (22). Liminality is a concept used in social anthropology and relates to “the expressions and the style of orderings, arrangements, and movements during a phase in the life of an actor, agent, entity, or a structure” (23). It has a temporal dimension, something in between two more structured stages, and relates to a structure with a focus on the unstructured, uncategorized, undefined, spontaneous relationships between statuses, roles, and offices in society (23). On the individual level, it refers to adaptation, and coping liminality (24) is the established theoretical concept used to explore how people may exist between stages and ages.

The aim of our article was to explore how now living Chinese old persons tell about their lives characterized by repeated periods of extraordinary and fast-changing contextual conditions. We also want to examine how theories of life courses can increase our understanding of such experiences and their impact on the individual agency. We will discuss what it could mean to understand the temporary as the permanent space in a life story. Our discussion is based on Settersten’s understanding of childhood as both a permanent and temporary space (25). According to Settersten, we need to take a long view of individual lives, although it is also relevant in understanding the collective lives of cohorts and other groups. What happens with our understanding if we identify the agreed temporality as a constant part of the structure and the agreed permanency as reduced to a part of an individual story?

## 2 Theorizing life courses

Elder (2) defines four concepts. (1) *Lives and historical times*: Individual life courses frequently serve as microcosms of the larger historical epochs they transpire within. Historical effects on the life course take the form of a cohort effect in which social change differentiates the life patterns of successive cohorts. (2) *The timing of lives*: Social timing refers to the incidence, duration, and sequence of roles and to relevant expectations and beliefs based on age. (3) *Linked lives*: Human lives are embedded in social relationships with kin and friends across the life span, and social support and behavioral exchange interfere with the life course. (4) *Human agency*: People make choices among options that construct their life course, and the life course implications of radical change, for drastic development and health, vary according to the personal experiences that people bring to new situations (2).

For our informants, the historical time was turbulent during large parts of their lives. In a comparison between China and contemporary Western countries, the differences in possibilities to plan the life span and to pass the passages intentionally are obvious. Nevertheless, there are historical events in the history of Western countries that can be used for comparison. The late-life situations of the cohort of what we studied cannot serve as a model for subsequent generations (26).

### 2.1 Stages of a life course

In demographic research, different stages that persons within a population should pass are described. In early life, persons are supposed to confront structures that socialize them into homogeneity. Pre-schools and schools for children and young adults into citizens make them more equal than in any other stage. In China, it was about the opposite. The social systems collapsed, and the chaos that followed created expectations that persons who passed their childhood and youth at that time would remember other events than those that may be associated with the current stage when they narrate their childhood. It is not just about their childhood experiences. It is also about a major structural change in society. When old Chinese people were young, the society was in a pre-transition life course stage. By the end of their lives, China had changed socially, economically, and demographically into what in Figure 1 [from Brown and Lynch (9) is described as a post-transition life course].

### 2.2 The continual transition into modernity

In the post-transition life course, there are two new stages related to modernity. An old person, still alive, can have been born into the pre-transition life course expectations and end life in the post-transition life course expectations. The two new stages, namely, education and empty nest and retirement (Figure 1), are especially interesting as our informants represent a generation who very compressed and, during their lifetime, have experienced what a change from pre-transition to post-transition of life courses can mean in the individual case.

The transition from being born into a society where you could expect a pre-transition life course to ending life in a society that has been transformed to a post-transition life course is not a question extensively discussed in life course literature. Few studies with a focus on external factors with a radical social change during the life course can be found. In Sweden, we found some exceptions. Sweden changed during the last century from being a poor country into one with an institutional welfare state, and Gunnarsson (27) found in narratives from people who had experienced rapid development, with migration into cities admission to longer education, and women’s entrance into the labor market. In men’s narratives, work was an important part of their life stories. In women’s lives, children were an important part of their narratives but also their work outside the home. Gunnarsson (27) found a group of older adult people, who experienced changes and are still active, with a strong feeling that life still is possible to schedule. Snellman (28) found that the women in her Swedish material who experienced a period of excessive female entrance into the labor market also chose to tell about their own experiences of that transformation and the consequences for attitudes and their links to family and others. They unexpectedly chose to underrate their everyday lives with their husband and children which took part during a longer part of their lives. If we follow Gunnarsson (27) and Snellman (28), we could expect to find that “the new” stages, *education* and the *retirement age,* should become exaggerated in the Chinese narratives.

### 2.3 Temporal breakdown of normal life

The Great Depression in the 1930s US is one historical time of interest for us. In a study, woman from the 1900 generation and their adaptation to 1930s conditions were selected (1, 29). Caspi and Elder (1, 29) conclude that adaptive personal resources and historical factors interact with social conditions, and also attempts to theoretically link class structure with personality were done (30). Ardelt (31) focused in her study on the relationship between the experience of economic hardship during the Great Depression and wisdom developed in terms of integration of cognitive reflective and affective personality characteristics developed in later life. She found that one path to wisdom is the successful resolution of crises and hardships. Crisis can lead to personal growth but is of course only one possible way to wisdom.

Another comparable historical period could be for persons born when the Great War broke out, entered the labor market, and established families during World War II. In a cohort of American men from age 18 to the late 30s entering World War II in their mid-20s or 30s, it was found that disruption in the life course caused a discontinuity in the line of work. Timing, how old they were when they entered the war, was important for their establishment in the labor market after the war service (32). A greater percentage of later entrants do show patterns of physical decline or death in the 1970s. A lack of communication and failure to talk about and share experiences leads to poor knowledge about such experiences.

A European cohort born during World War II differs from other cohorts in various respects citizens during 6 years were subjected to battles and bombing, they were forced to abandon and give up property without compensation, they had to move from their lands, they experienced hunger and were separated from their families, and many children lost their fathers. The long-term health outcomes were a higher probability of diabetes and depression. The chances of getting married were reduced for women and the opposite for men. In all, those citizens had less education and less life satisfaction than citizens in European areas who had not lived in war countries and were exposed to combat (33). Kesternich et al. (33) have, through using relevant variables, namely, mortality, sex ratios and absence of father, hunger, dispossession, persecution, and migration, given a valuable step forward in the understanding of how people can be differently affected by events which took place during the same period and sometimes in the same country.

One focus in the Western discourse is on how attitudes are transformed within quite stable families (34). Both the AJW and the CR caused the loss of family members or split families.

Most sociological work on the life course and large-scale social change concludes that a breakdown of the normal life course tends to produce negative effects on subsequent life experiences. Amazingly Zhou and Hou (19) find that the lives of sent-down youth were not as damaged as one might have expected. The authors found that if the sent-down youth stayed in rural areas for less than 6 years, their mid-life income was not significantly different from the income of those without send-down experiences, and the sent-down youth reported much higher proportions of entering college and holding high social status in China as cadres in government. This finding may lead to a significant theoretical modification of the life course approach—that is, the interference or impact of state policies on individuals’ “normal” life course could be negative or positive or even both (e.g., short-term negative with long-term positive consequences). Zhou and Hou (19) suggest possible adaptations. First, getting used to tough physical labor was their first priority. They then had to strive for upward mobility in their rural lives and in their dreams of returning to the cities. On the one hand, the sent-down youth may be more adaptive as their unusual experiences probably improved their social skills and increased their political flexibility. On the other hand, some adaptations learned in the sent-down years would not necessarily be helpful strategies and might even be hindrances, in successfully adapting to the reform years.

Settersten (25) concludes that there is some agreement about which factors old people have prospects of failing health or chronic health conditions; greater salience of health concerns in individuals’ self-definitions; diminishing time left to live and the need to come to terms with one’s mortality; bereavement associated with the death of parents, spouses, and friends; more restricted but intense social relationships and networks; being perceived or treated by others in ageist ways; increasing interiority, desire for integrity, and search for meaning in life; and greater acceptance of things that cannot be controlled in life, coupled with greater fear of losing control over one’s life. Settersten gives those factors a status of temporality, underscoring their dynamic nature as they evolve over the course of one’s life (35). What happens if we give those factors a permanent place in life from childhood to old age?

### 2.4 Chinese studies with a focus on health in later life

In some Chinese studies with a focus on health in later life, it was found that there is an important correlation between education and health in later life. Lou et al. (36) first found a negative correlation between education and mortality, but when other socioeconomic attainments were added the correlation was more blurred. The positive education effect on mortality was more associated with *social relationships and leisure activities* than with smoking and drinking. One study revealed the impact of important life courses such as retirement, widowhood and loss, and the older adult population on the mental health of older people based on the life course-ecosystem model (37). We take note of this result and continue to search for other underlying social factors that explain success in life. Fan (38) investigates individuals’ decision-making processes pre-, during, and after CR, for example, how to predict school reentry. The study shows how policy-changing institutions intersect with individual behaviors to shape educational strategies over time. Shen and Zeng (39) found that those who *survive extreme experiences* in childhood are more robust and have lower mortality in advanced ages (40). Young and Lou (40) studied the relationship between childhood adversities and depression. The people from the traumatic childhood class (who had experienced famine, a mother’s death, and a father’s death) reported the lowest depression score, while economic problems (parental illiteracy and no schooling) in childhood were not as discriminating. They suggest that this result has to do with a selection effect, and those who survived traumatic events also can *cope with life stressors* better. Another result is that a parent’s death not necessarily meant limited resources as the joint family was an important family type when those persons were young. Education was an important preventive factor against depression. Wei Huang and Yi Zhou (41) found that the Great Famine can result in long-term health consequences through the pathway of losing educational opportunities rather than through the pathway of nutrition deprivation The researchers asked for more data on life adaptation and resilience and protective factors such as personality traits, peer affiliation, and *problem-solving skills*. This study takes an important step into the definition of contextual factors which can help understand how people experience and narrate their life course. We can summarize that human agency seems to be important in social situations defined as liminal.

The need to question the dominant position of biomedical discourse is formulated by Kwok and Ku (26) who have studied a Hong Kong cohort of older adult people. A just description of the life experiences where the researchers look back and consider the contributions that this cohort has made to society is important to develop a genuine self-understanding of the specific society. One weakness in many of those studies is that age is not problematized. Often all person 45+ is classified as old, and the questions about, for example, the experience of famine are not linked to a specific historical time (40). Huang and Zhou (41) who studied the effects of the GF discuss the methodological problems with this discontinuity in the continual development. The cohorts of 1948 to 1953 became problematic and deserved a special methodological solution, namely to copy persons born in 1948 to substitute those who were born in the following 5 years.

## 3 Methods

This study uses an interpretive paradigm and qualitative research methods. The research team collected life story materials of Chinese older adult people through in-depth interviews which were conducted in Jinan City, Shandong Province. Selecting Jinan as the research site is not just because it is where the research team lives and works, which facilitates the conduct of the interviews, but also because more importantly, it is a representative Chinese city which is located in the middle of Shandong Province and has a high level of aging, with about 16.9% of the total population over 60 years old. The older adult people living in Jinan are not only original city residents but also those who were born in rural areas and later went to school and work in Jinan and those who moved to Jinan to live with their children after getting old.

### 3.1 Data collection

A purposive sample consisting of 16 Chinese elderlies was interviewed in Jinan City, Shandong Province, China, at the end of 2012. They were asked to tell their life-starting stories from their first memory. Master of Social Work students at Shandong University collected life histories from old persons.

As interviewers, the MSW students were equipped with a list of possible questions to ask related to memories from the different phases of life, their relationships in and outside family, their working life, and life as retired. They could use that list relatively freely, and it was the informant who had the power to decide how the story should be told. The students used, to a limited extent, the list built on a notion of how a standardized biography could be built up. The stories were formulated from a non-standardized script (42).

*Ethical consideration*: The informant would not be someone whom the student was already familiar with. The autonomy of the informants was totally protected as no names were registered. If a selected person did not want to participate, the student was instructed to find another informant who agreed to participate, and they were all informed that their responses should be discussed in the class without any identification mentioned. That promise created a confident relationship.

The basic information of 16 older adult people interviewed is shown in Table 1, from which we can see that the informants who were selected as interviewees were all 61–75 years old in the year they were interviewed. Of the 16 informants, 8 are male informants and 8 are female informants, each accounting for 50%; 8 are urban residents and 8 are rural residents who were currently living in cities, each accounting for 50%. They were born from the late 1930 to 1951; among them, 6 were born in the 1930s, accounting for 37.50%, 9 were born in the 1940s, accounting for 56.25%, and 1 were born in the 1950s, accounting for 6.25%. Many of them describe their earliest memory from the AJW that took place between the years of 1937 and 1945. As adults, often when their own children were small, they experienced the CR which took place from 1966 to 1976 and that means that they have experienced two stages where the predictability of what was to come in life was reduced.

**Table 1:** List of informants.

No.	Gender	Age	Year of birth	Region	Occupation	Years during AJW and C W	Age when GF started 1959	Age when CR started 1966
1	F	63	1949	R-U	Accountant	Born after	10	15
2	F	73	1939	U	University Teacher	10	20	25
3	M	64	1948	U	Worker	1	11	16
4	M	71	1941	R-U	Educator	8	18	25
5	M	71	1941	R-U	Farmer	8	18	25
6	F	70	1942	U	Worker	7	17	24
7	F	63	1949	U	Accountant	Born after	10	15
8	M	74	1938	R-U	Professor	11	21	28
9	M	75	1937	R-U	Teacher	12	22	29
10	M	66	1946	R-U	Farmer	3	13	19
11	M	72	1940	U	Farmer	9	19	26
12	F	74	1938	R-U	Nurse	11	21	28
13	F	61	1951	U	Worker	Born after	8	15
14	F	74	1938	U	Civil servant	11	21	28
15	M	72	1940	R-U	Worker	9	19	26
16	F	74	1938	U	Doctor	11	21	28

### 3.2 Data analysis

We utilized NVivo 12, a robust qualitative analysis software renowned for its extensive capabilities in coding, summarization, and data visualization. This software proved instrumental in enhancing the precision and efficiency of our analysis, thereby ensuring the objectivity and authenticity of our study results (43, 44).

In this research, we employed grounded theory (45) to condense, compare, conceptualize, and synthesize patterns within the collected data, approaching the investigation with a “naturalistic” perspective. The coding analysis encompassed three primary stages:

1. In the initial open coding phase, we imported 16 interviews into the NVivo 12 software and meticulously reviewed and extensively open-coded the source materials. This process yielded 21 initial concepts and 643 informative references pertaining to the life course and significant life stages of older adults.
2. Subsequently, in the axial coding stage, we distilled the initial concepts and reference points identified during open coding, delving into the inherent logical relationships among these concepts. This exploration led to the extraction of core themes that unified various sub-themes. For instance, themes such as “loss of family members,” “interruption of education,” “poverty and starvation,” and “categorizations in order to split the social relationships and networks” emerged.
3. In the final selective coding phase, we refined and organized the existing themes and sub-themes, presenting the life course and a chronological framework of different interviewees’ life spans. The study delved into the implications of early life disruptions on social identities and relationships in adulthood within the context of life course theories.

## 4 Results

### 4.1 A preliminary analysis

Although the interviewers’ list of possible questions was conventionally structured, the informants chose to tell their lives based on the dramatic historical events, the AJW, and the CR. The informants experienced the up and down turbulence, rapid development, and intense social transformation of China in the last 70 years. In the narratives, the fast-changing contextual conditions, and the development from a poor and pre-modern society to a rich and developed society, seem to be taken for normal, and they tell about a happy later life.

#### 4.1.1 Linked lives

Most informants were born into big and varied families with many siblings. No 1, born 1949, was the third of four siblings born in a village; No 2, born 1939, had several siblings as her father had many wives; No 7, born 1949, was married to a man with six to seven siblings; No 11, born 1940, is the fifth of six siblings; No 14, born 1938, had one brother and six sisters. Her parents’ desire for more sons was the reason for the great number of children. Normally, the informants had lost close family members by starvation or war activities, which have either strengthened or weakened family bonds.

The year of birth makes a big difference. Some of our informants had experienced all the periods of turbulence, while others, only a few years younger, had missed the tumult during the early 1940s or even the CW. If the tumult happened during childhood when they still were dependent on family members for survival, the tumult will have another meaning than if they were parents and/or wage-workers and as such citizens with responsibility also for others when it happened.

### 4.2 Anti-Japanese war (1937–1945) and civil war (1946–1949)

As expected, the narratives have similarities with those reported by Kesternich et al. (33) from World War II. Early childhood was, for most of our informants, full of suffering. For informants who were born in the late 1930s and early 1940s (such as 2, 4, 5, 6, 8, 9, 12, and 16), their early memory emanates from the AJW and succeeding CW.

**Theme: violence**

**Subthemes: family communication, escape, being robbed, and hijacked**

The first memory of one of the old women (No 6, born 1942) was from the war. Japanese robbers came into her house where she and some friends played together. A bomb was detonated. Her aunt protected them, and the robbers left. When the country was about to be liberated, the children were sent out to rob something. She did not obey, and her mother complained about that. At that time, she had no dreams. Her story is a common description of life at that time.

Early experiences were in the narratives related to the loss of family members, fear and unstable life, escape, and interruption of education. Some events our informants tell about are stories from their childhood told by other family members: *My earliest memory is that I was told that I was born in 1938 when there was AJW* (No 16, born 1938).

*I was told that when I was around 7-8-month-old, a group of Japanese soldiers came to my house and took me away which scared all my family. But they sent me back afterward. I thought that the racketeer might miss his own child who might look a bit like me. He not only made a photograph of me but also gave me some candy* (No 16, born 1938).

*Similar to No 16, born (1938), many families were fleeing. Several stories are about canceled or absent school life. “Actually, I did not go to elementary school for a very long time, because we fled as a refugee when the AJW started”* (No. 2, born 1939)*. The stories often include dramatic elements. “I started elementary school when I was 6 at the same time as the eve of the New China’s birth. I hid in the landlord’s house when the People’s liberation army attacked the Kuomintang Army in Jinan City. I restarted my education after the liberation”* (No 4, born 1941)*. “A group of Kuomintang soldiers intruded on my house and tried to find valuable things and hit my aunt with a gun, something which made me scared and cried”* (No 6, born 1941)*. “My father was kidnapped by bandits who claimed money from my family”* (No 12, born 1938).

**Theme: family and linked lives**

By exploring how individuals are connected through interactions with family and friends, continuities and discontinuities can be revealed. Our informants tell about murdered family members and family members who disappeared.

**Subthemes: loss of family members, absent fathers, and unstable life**

*My grandfather was murdered by the landlord's restitution corps in 1948 years* (No 8, born 1938)*. The men were taken out for military service, and the women had to stay home. “I couldn't get my father's care when I was a young child. When I was two years old, when the AJW was going on, my father went to join the People’s liberation army to fight Japanese imperialism. My mother raised us all by herself”* (No 9, born 1937)*. Even if the fathers operated nearby, the relationship could be absent. “I never saw my father and didn’t know what he looked like. Before I was born, my father went to join the army and became a spy of the CCP. My father dared not show himself publicly, and always came back home after dark and left home before daybreak”* (No 12, born 1938)*. “My father was the only son of my grandfather and was also the youngest among his siblings. After being released, he was sent to the army of CCP”* (No 12, born 1938).

Those children had already experienced what Settersten (25) defines as a “characteristic of old age”: mourning associated with the death of parents, siblings, and friends, and maybe the understanding that death is constantly present. Furthermore, they must acquire a fatalistic attitude as life is unforeseeable something that also creates the need for increased vigilance.

### 4.3 The great famine 1959–1961

Those who had a long experience with the AJW and the CW such as No 8, No 9, No 12, No 14, and No 16 were in their phase of family formation when the GF broke out and even older during the CR. There are three persons (No 1, No 7, and No 13) who were born after the AJW and the CW, and one person (No 3) who was only 1 year when that period ended. Those persons (and also No 10 who was 3 years when that period ended) could be expected to have other experiences than persons who were adults during that period. Some of our informants had not reached their teens (No 1 born 1949, No 3 born 1948, No 7 born 1949, No 13 born 1951), while the other informants had already created their own families and had small children.

**Theme: poverty and starvation**

Most of our informants have mentioned their experiences of hardship and poverty when they were children and young adults: lack of food and other necessities of life, lack of money, only having poor clothes, etc.

**Subthemes: food, clothes, and housing**

From many stories, we understand how close they were to dying. Not having enough food and having no high-quality food are their common and impressive vivid memories. Almost all the selected informants mentioned such experiences. “*There was almost no money and there was no nutritious food, it was hardly we had enough food to eat. Rural people were not submerged in agricultural cooperatives when I was a child, there was not enough food for my family, and we could not eat well, sometime we could only eat soybean curb residue, so I admired families who could eat well”* (No 1, born 1949). “*In 1960–1962, sometimes we could only have leaves to eat”* (No 11, born 1940). And “*We were not allowed to eat as much as we needed, only guests could be served steamed buns made of wheat flour which we could hardly have chance to eat”* (No 14, born 1938).

To find food was then of the highest priority. “*When food was very limited, surviving was the first concern, no one cared about clothing. Everything valuable was sold for money which could be used to buy food in rural areas* (No 3, born 1948). *We could only have dumplings once a year when celebrating Chinese New Year”* (No 4, born 1941).

They also tell about outdated equipment in the households. “We *still used kerosene lamps to light and still ate steamed corn bread and pickles”* (No 4, born 1941). And they had to beg for food “My *mother often brings me to the village head’s house to ask for food”* (No 9, born 1937).

Help from the community was not sufficient.

*“Our factory was a partner of a people's commune in Feicheng, a county of Shandong. They provided leaves of sweet potato to eat. I also had eaten poles and leaves of lotus, roots of grass, etc. At that time, factory life was even worse than life in the countryside”* (No 11, born 1940).

And the people fled because of hunger.

*“You could see that all leaves of trees were eaten out. White clay to ease was also called Kaolin or amosite, when there was no food to be eaten, people could only eat white clay to ease. (…) When people eat too much white clay to ease, they may die. But people had to eat white clay to ease because there was no food and other things to be eaten then. People went everywhere to flee from famine”* (No 15, born 1940).

Some participants described their tough experiences with clothes when they were children and young adults.

*…, life was hard … You can see in the photo that my shoes are broken and the cotton in them could be seen …Just after the New China was born, life was not well for everyone* (No 3, born 1948).

*…There was no money to buy new clothes for four kids even in Chinese New Year. I did not want to visit my parents if there were no new clothes for each of my kids, so I often fight with my husband in New Year at that time* (No 6, born 1941).

A more elaborate version: *Everybody was almost equally poor, so people did not feel hard* (No 12, born 1938).

Not many participants mentioned their poor housing conditions and other sides of life, but that does not mean they lived very well then. The poverty continued for a long time, and this person talks about the situation in the 1970s.

*My husband came back on vacation from military service one winter, it was very cold and there were not enough quilts to cover when we slept. The house had cracks, when it snowed the snowflake could be scraped into the house. We covered everything we could use to keep warm when we slept and survived that* (No 1, born 1949).

Another informant describes how they gathered things as fuel to cook food which can be an indicator of the hardship of life at that time.

*“At that time, it was really very poor. In autumn, after harvest, people went to land to collect roots of corn, and in winter, people went out to pick geese droppings. These all could be used as fuel. How suffered I was in the factory, your young generation had never experienced”* (No 11, born 1940).

Our informants have now passed two serious national catastrophes and are in the phase of family formation and entering the labor market. Our informants are all survivors, used to a life that social anthropologists should call liminal, and defined as uncertain, unstructured, and unpredictable.

### 4.4 Cultural revolution 1966–1976

The informants, during the CR grown-ups and between the ages of 20 and 30, told something about their experiences of CR. Those narratives are different compared to those about the AJW. Our informants’ reflections now include a social-political dimension which they were not able to include in their memories from their childhood. The stories they tell about a society where their attention is drawn to social differences and inequalities.

During the CR life, people worked in productive teams with limited control over their personal lives. A woman (No 6, born 1942) remembers how she worked hard and many hours in the fields. She had no time to rest. When the leader said it is time to work, she had to do so. She had no time to take care of her child. Once her neighbor contacted her and said that her child had fallen from the bed, first, they did not find the baby, but when they found him, he had laid himself under the bed and fallen asleep.

**Theme: categorizations in order to split the social relationships and networks**

The stories include massive violence toward family members. In their stories, they give examples of their problem-solving skills when they have to cope with life traumas. This woman and her son survived thanks to her potential to live from hand to mouth and to create new and helpful relationships. “*There were indicators then: there are 95 % of the good guys, and the other 5 % are bad guys. People fear being categorized to be ‘Bad guy’ of that 5 %, so everybody should be able to recite Quotations from Chairman Mao*” (No 15, born 1940).

One 73-year-old lady (No 2, born 1939), who had earned her living as a university teacher, tells the following: “*In the CR, I was forced to ‘Laogai’-reform (recorrection) through labor (hard work) which was regarded as punishment for us who were considered to be opposed to socialism, but I thought it was a chance to learn from other people under labor reform (recorrection through hard work).”*

This lady, born into an upper-class family with a father who was once a Kuomintang military officer and an artistic mother, was classified to be on the enemy side. The subtheme of *violence* which we frequently found in the stories from their childhood also came up here:

*“They beat me deadly, whipped me, tortured me, and made me black and blue all over my body. (…) It was not easy and a kind of luck to survive. My mother living in Beijing was during the CR forced to go back to my hometown. She was very sad and died in my hometown. (…)I suffered endless bullying. were separated by force from my family”* (No 2, born 1939).

*Violence was also directed toward her son “In the CR, some people discriminated against my child”. One primary school teacher always tortured my son because he saw everything through colored spectacles. Some of his classmates said to my son “your mother was a rightist who was anti-Party and anti-socialist”* (No 2, born 1939).

**Subthemes: linked lives, continuity, discontinuity (the people), discontinuity (enemies), and timed opportunities**

For those who were on the “right side,” life continued like before, as for No 8 (born 1938) who worked for the army after having completed technical school, or No 7 and No 10. A woman who later became an accountant told: “*The CR had not much effect on us; (…) there would have no effect on you if you had ‘red’ family background*” (No 7 born 1949). Or this farmer: “(…), *the whole country was in a crazy situation, my family was of peasant origin, and had not been much affected and could live a fairly normal life”* (No 10, born 1946). For some, it meant less education as schools were closed as for No 7 who also said that CR did not affect the opinion. “*We just stayed at home, could not go to school anymore. There were no schools all over the country. We, students, played day by day. Some people traveled from place to place rebelling against those in power and making revolution just for fun, after that, I went to work”* (No 7 born 1949).

To take part in the ongoing social change could be a free choice, such as for No 9, born 1937, a person born in urban areas and was 29 when the CR started, used his agency to follow the call from the Prime Minister to form a new society:

*“I was affected by the CR. The main reason was I grew up in Qingdao, a big city in Shandong which was a more developed area in Shandong. I could get a job in Qingdao, but when I graduated from university, Premier Minister Zhou Enlai gave a speech that called us ‘Go to the border, Go to the countryside, Go to the places where you are most needed by motherland’. I saw a movie that encourages youth to devote themselves to the development of the countryside. I was inspired by Zhou’s speech and the movie and decided to go to the countryside instead of staying in Qingdao. The 12th middle school where I taught was located in the mountain area, even local people were reluctant to teach there which was isolated from the outside world, as a person from other places I didn’t know that before I went there.”*

This informant later worked as a teacher.

For those who were classified as enemies of the people, it meant the death of family members, prison, broken family ties, hard work in a countryside that they did not know, and hard work that they were not familiar with. For those who lived in cities, it could mean that they had to move to the countryside and learn from the people through hard work. “*I even did not get the news about my mother’s death at that time. Everybody suffered a lot and could not get messages from each other easily. My family was very miserable at that time, some family members died of hunger and some family members were killed”* (No 2, born 1939).

Those who grew up before CR had more foreseeable opportunities to get an education compared to those who grew up during CR. No 4 born 1941, who above told about his interrupted schooling, later became a teacher and went to university before CR, but his younger brothers and sisters could not enter higher education because the schools and universities were closed due to the CR.

*“My siblings envied me because I went to university, and they lost the opportunity to get high education because the CR. I entered university in 1960, and graduated in 1964, not affected by the CR. None of my siblings couldn’t go to school, couldn’t go to university, and because they didn’t go to school, they became temporary workers and workers”* (No 4, born 1941).

## 5 Discussion

According to the stories, the memories from the series of structural breakdowns dominated their childhood and early lives. The unstructured, discontinuous, and unpredictable have tended to characterize the normal childhood and adult life, realities that in the West are associated with temporality and deviation. *Normality* then becomes a concept for further exploration in relation to how people perceive social conditions and present their life histories.

One important finding is that to make it possible to study causation in a fast-changing order, the groups studied must be categorized in short age ranges. In the personal adaptation to historical factors, the year of birth can be decisive. To be in school age before or after the CR made a difference. To be born before a certain year could mean access to education, while another year of birth does not. The *timing* was then very visible in people’s lives. A concept such as linked lives as embedded in social relationships with kin and friends across the life span becomes problematic in the Chinese pre-transition life course (see Figure 1) framed by war and national upheavals. Information about family relationships was limited unless the story did include shocking memories of violence, escape, or starvation caused by factors outside the family’s control. In the material, we found broken links and such disconnections early in life which is unexpected according to dominating west-impregnated life course theories. The stories include many examples of how the informants lost close family members during the war, during the GF, and the CR. These breaks in relations were not the result of an individual decision but the result of the unreliable structure in which they spent their childhood. Their families had limited possibilities to decide how social links should be continued. According to Young and Lou (40), our informants belong to a cohort well-trained to cope with life stressors. Alternatively, you can imagine that they harbor significant cohort memories of their generation and should have long-lasting effects on them.

Another finding is that unpredictable and disruptive change has a significant impact on the educational phase of an individual’s life course, which would have an important influence on their later work and life. Education, the stage following childhood in Figure 1 we presented in the introductory part from Brown and Lynch (9), became, as expected, for many an important part of their story. For some, it was presented as a path to a better life chosen by themselves, and for others, the education period, such as for example No 2 and No 4, was a lost period in life. In some families, only some children had access to education. The schools were closed when the other children were of school age. Interrupted education caused a discontinuity in many lives. No 4 became a teacher, while his siblings became blue-collar workers. In his story, that was a reason for jealousy between siblings and perhaps later difficulties in maintaining family relationships.

If we shift the focus to what happened in parallel to general modernization, we find that the value of education changed over time. Our informants all were born in a historical time when for most people the transition to adulthood probably was as apprentices (see Figure 1). Formal education had not been as necessary for their later position in society. For some of our informants, there was no problem that the schools were closed, for example. No. 7. During their lifetime, they might come to experience how formal education becomes an important and maybe necessary part of a normal life course.

Our interviews show that an age difference of a few years could determine whether they could get an education (before CR) or whether they were stopped in their educational ambitions (during CR). Some informants tell about frustrations over injustice which could mean that persons having a high education before the CR were not well treated in the CR while after CR there was a shift in the opinion and education had a positive effect on their lives and their possibilities to improve their opportunities in life. The shift from an ambivalent attitude to formal education that our informants have experienced, from something for the elite to being a burden, and finally to something for the masses they tell about in their later life may be more explored to give a deeper understanding of how rapid changes can be justified in people’s minds.

Similarly in the stories from WWII (32) about labor market entry, we can talk about *timing* as a very tangible and concrete phenomenon. This link between lives and historical times manifests in that the relation to education is an important part of how many stories were structured. The latent story of constant modernization was not told. In contrast to Gunnarsson (27) and Bernardi et al. (46), we found it complicated to separate the contextual factors from the everyday lives of the informants.

Through these cases, we also think about how interruptions in important life courses can influence future life courses. Brown and Lynch’s model (2015) of life stages in the post-transition societies introduced in Figure 1 includes another new stage that belongs to modernity, the stage of *empty nest and retirement*. Happiness and wellbeing linked to leisure time as a factor preventing depression in later life are an important theme in gerontology research (47, 48). Although this exploratory study is based on a small sample, we can still imagine what a large part of China’s aging population has lived through and how they might have experienced it. According to life course theory, an individual’s experiences at an early age can influence behaviors at a later age, thereby creating continuity throughout the life course. This case raises a number of questions about how a society with an overwhelmingly large group of older people is affected by their experiences and how they are passed on to the next generation (49).

In Western societies, aging often means that people adapt to declining bodily functions, declining financial resources, and a reduction in social networks. Life satisfaction seems to remain stable during a lifetime (14, 50). For this study, the adaptation must take another shape, from extreme poverty to an in many characteristics better life. In Western societies, there is an expression saying “Life was better before” which for an old person could be interpreted as “Before my health was better, many of my relatives and friends were alive and I was active in many different arenas,” but for a person who during their lives have experienced the losses of family members, had bad health due to starvation, and lack of warm clothes and the public arenas were dangerous to visit. We can understand that they have many reasons to compare their present life with earlier stages and say “Life was not better before.” In this study, we have chosen not to focus on health effects but rather on human agency and skills to survive extreme situations.

The material invites us to further reflect on how people use their *agency* to adapt personal resources to historical factors and social conditions from earlier to the present time. One of our informants, No 7, choose to become a Red Guard and used his agency to follow the leader, while the others either choose to describe themselves as victims or as not involved in politics. Many of them, such as no 2 (born 1939), gave us many examples of problem-solving skills that she had not been familiar with. They can choose to construct their life courses subjectively so that the different parts of life become meaningful in their narrated life story.

Our research has the following significance; first, it enriches the life courses theory and provides a new perspective for us to study the individual life course in a society of constant and rapid change. Second, it provides a direction for us to understand the impacts of wars, famine, and drastic social reform and revolution on the individual life course. Finally, it is an important reference for the study of the life course of a particular group in other countries that have experienced successive wars or other major changes in their societies.

### 5.1 Need for further research

In Western societies, the possibility to plan one’s life is more or less taken for granted. We have shown a material where nobody was able to plan their lives in that meaning. Our material has awakened an interest in using relevant comparisons of historical events (33, 51) to develop life course theories.

When interpreting data on life courses from a non-Western country, we found some thought-provoking assumptions in the theory. The thoughts of stability and continuity are one. The temporal seems to have been the norm in China for long periods. There is a need for further research on liminality, the socially unstructured, the unexpected, and even the chaotic in people’s lives that have an impact on their social links and identity formation in later lives.

Another concept to be researched is *choice biographies* (52, 53) in contrast to standardized biographies and that means dis-embedding of individual lives from the structural fabric of social institutions and age-specific norms may be a method to visualize a continual transition.

## Data availability statement

The original contributions presented in the study are included in the article/supplementary material, further inquiries can be directed to the corresponding authors.

## Ethics statement

The studies involving humans were approved by the Ethics Committee of Shandong University. The studies were conducted in accordance with the local legislation and institutional requirements. The participants provided their written informed consent to participate in this study.

## Author contributions

S-LC: Funding acquisition, Project administration, Resources, Writing – original draft. SJ: Conceptualization, Investigation, Supervision, Writing – original draft. SL: Data curation, Validation, Writing – review & editing. YL: Software, Writing – review & editing.
